# Regeneration of corneal endothelium following complete endothelial cell loss in rat keratoplasty

**Published:** 2010-11-11

**Authors:** J. Schwartzkopff, L. Bredow, S. Mahlenbrey, D. Boehringer, T. Reinhard

**Affiliations:** University Eye Hospital Freiburg, Killianstr. 5, 79106 Freiburg, Germany

## Abstract

**Purpose:**

Corneal endothelial cells (EC) are crucial for maintaining corneal clarity before and after keratoplasty. Since it is thought that corneal graft rejection leads to irreversible EC loss and transplant failure, we quantified immune mediated EC loss in the rat keratoplasty model and analyzed whether the EC layer would then regenerate.

**Methods:**

Rats were subjected to orthotopic penetrating keratoplasty. We compared endothelial responses to immunological EC loss following allogeneic transplantations between Fisher and Lewis rats (group R) to those following mechanical EC removal in a syngeneic setting between Lewis rats (group S). Animals were followed clinically for corneal opacity for up to one year. Bulbi were excised and prepared for histological examination at different time points: ECs were defined and characterized using Alicarin red S/ DAPI staining on corneal flatmounts. Ki-67/ DAPI staining on flatmount preparations served to detect cell proliferation. Immunohistochemical staining of corneal cryosections was used to characterize infiltrating immune cells.

**Results:**

Group R: After about two weeks the allografts were completely opaque, which was accompanied by a massive leukocyte infiltration in conjunction with EC destruction, signifying rejection. EC loss without an immune reaction (group S) resulted only in medium opacity levels. In both groups, all grafts regained clarity in the following weeks to months, and a newly-formed endothelial cell layer with irregular and enlarged ECs became apparent on the formerly EC free grafts. Scattered Ki-67 positive cells within the endothelial cell layer were observed during re-endothelialization. In addition to re-endothelialization, the immunological infiltration seen in the allografts at the time of rejection had subsided after one year.

**Conclusions:**

Re-endothelialization following keratoplasty takes place in the rat in vivo and restores graft clarity, following both immunological or surgical destruction of ECs. Following rejection, EC replacement is accompanied by a reduction of immune infiltrates. Peripheral recipient ECs are a sufficient source for graft re-endothelialization, as seen in rats following EC removal. Our results suggest that ECs both proliferate and enlarge during re-endothelialization in the rat keratoplasty model.

## Introduction

A clear cornea is essential for good visual acuity. This requires an intact corneal endothelium, which actively dehydrates the corneal stroma and thereby prevents corneal edema and concomitant opacification. Since this cell layer does not regenerate in vivo in humans, a constant and age-dependent loss of corneal endothelial cells occurs. Any form of corneal damage by inflammatory processes or by mechanical trauma following intraocular surgery or penetrating injury can lead to additional endothelial cell loss. Once the endothelial cell (EC) density falls below a critical number, corneal decompensation follows. In such cases, transplantation of a full-thickness corneal graft or isolated endothelial cell layer is the only therapy to restore corneal clarity in the long run. It is also imperative thereafter to maintain a high endothelial cell count on corneal grafts to guarantee corneal clarity.

Physiologic EC loss is accelerated further following penetrating keratoplasty (PKP) [1-4]. Surgical trauma or immunological factors leading to accelerated loss of corneal endothelial cells are discussed in this context. Following lamellar endothelial keratoplasty (DSAEK), an increasingly popular technique [5], EC loss seems to be even more pronounced than following PKP [6,7]. Therefore, better understanding of the mechanisms of EC regeneration following keratoplasty is an important, topical matter of concern.

Today, evidence suggests that at least some endothelial cells are capable of dividing and proliferating in humans as well [8-12]. According to in-vitro assays, these cells appear to reside in the corneal periphery primarily [13]. Still, the knowledge regarding mechanisms and dimensions of corneal EC regeneration in vitro and in vivo is limited. Endothelial regeneration in vivo has mainly been studied in corneal injury models such as corneal freezing or scraping. There have been reports that corneal ECs in the rabbit, cat or rat can migrate and to some extent proliferate to cover defects [14-17]. However, surprisingly little is known about endothelial reactions following corneal transplantation. While it is commonly assumed that rejection leads to permanent EC loss, some authors reported restoration of corneal clarity following rejection [18,19], which would require a functionally intact endothelial cell layer. Despite these interesting observations, the extent and mechanisms of EC regeneration in vivo are poorly understood. A better understanding of corneal EC regeneration is necessary for investigating new therapeutic approaches such as raising endothelial cell counts for corneal transplants before surgery, enhancing tissue engineering techniques for the ex-vivo cultivation of endothelial cell sheets, or mechanisms to decelerate endothelial cell loss following surgery.

The rat keratoplasty model is widely used to study immunological responses following keratoplasty. Most authors analyze mechanisms that lead to rejection, or seek therapeutic strategies that prevent allograft failure. In these studies, analyses terminate with the onset of rejection. Opacification is generally equalized with rejection, and complete endothelial cell rejection or loss is assumed. However, few investigations have focused on the actual endothelial response following penetrating allogeneic keratoplasty itself. We therefore investigated the regenerative capacity of corneal endothelial cells in vivo in rat PKP, and assessed EC regeneration following immunological and surgical EC loss.

## Methods

### Groups

To analyze potential differences in endothelial reaction to EC loss due to rejection (immunological cause) or mechanical cell loss (surgical procedure), two experimental groups were analyzed (“group R” and “group S”), and compared to controls (“control group”):

Group R (rejection group); Allogeneic corneal transplantations were performed using Fisher rats as donors and Lewis rats as recipients. In this rat PKP model, a minor mismatch leads to a 100% rejection rate, which is thought to be accompanied by EC loss. Group S (surgical group); A corneal transplant without endothelium (mechanical endothelial abrasion before transplantation) was syngeneically grafted using Lewis rats as donors and recipients, respectively. Control group; Syngeneic transplantations with intact endothelium served as controls.

### Animals

All animals were handled in accordance with the ARVO Statement for the Use of Animals in Ophthalmic and Vision Research. Fisher and Lewis rats (>8 weeks old) were obtained from Charles River, Sulzfeld, Germany.

### Corneal transplantation and definition of graft rejection

Orthotopic corneal transplantations were performed as previously reported [20]. Briefly, animals were anesthetized by isoflurane inhalation, followed by intraperitoneal injection of a mixture of Ketaminehydrochloride 100 mg/kg, Atropine and Xylazine 0.5 mg/kg. Before surgery, mydriatic eye drops were applied topically. Donor and recipient corneas were trephined with a 2.5 or 2.0 mm trephine, respectively. In group S, the donor corneas’ EC layer was mechanically abraded with a hockey knife before transplantation. The graft was fixed with 8 interrupted sutures (11.0 Ethilon; Ethicon, Norderstedt, Germany). We then administered a tarsorrhaphy, which remained in place for the first three postoperative days. As previously described, all grafts were repeatedly analyzed for corneal opacity according to an international score [20]. Corneal graft opacity was graded as follows: 0: completely transparent corneal graft; 1: slight corneal graft opacity, but details of iris vessel easily visible; 2: moderate corneal graft opacity, iris vessels still visible; 3: strong corneal graft opacity, only pupil margin visible; 4: complete corneal graft opacity, pupil not visible. Rejection was defined as complete graft opacity (grade 4). Animals with surgical complications such as intraocular hemorrhage or cataract were excluded.

### Histological examination

#### Cryosections

Bulbi were enucleated and frozen in liquid nitrogen at different time points (day 0, day 15, 6 weeks, or one year following keratoplasty). To detect immunological infiltration of the cornea, cryosections were stained using the Streptavidin-AP (DAKO, Glostrup, Denmark) method. Primary antibodies against CD4 (clone W3/25), CD8 (clone OX-8), CD25 (clone Ox 39), CD161 (clone 10/78), CD163 (clone ED2) and for dendritic cells (OX62) were used (all from AbD Serotec, Kidlington, UK). Negative controls were performed using isotype antibodies (mouse IgG1; BioLegend, Uithoorn, Netherlands). Biotinylated rabbit-anti-mouse IgG was used as secondary antibody (DAKO). To perform cell counts, pictures of three microscopic fields in the superior third of transplants in the central region of the graft were taken and analyzed using a microscopic camera (Carl Zeiss, Jena, Germany) and analysis software (Olympus, Hamburg, Germany). All evaluations were performed by two independent investigators blinded to the experimental group.

#### Flatmounts

To assess the endothelial cells, bulbi were enucleated and the corneas excised. Flatmounts were prepared with the endothelial side facing upwards and stained with Alicarin red S for 1.5 min (pH=4.3) and cell nuclei were counterstained with DAPI (4′,6-Diamidino-2-phenylindole; Sigma-Aldrich, Munich, Germany). Cell density was evaluated in the transplant’s central and border areas as well as the peripheral recipient cornea (Carl Zeiss microscope and ‘analySIS’-software, Olympus).

To detect proliferating cells, corneal flatmounts were fixed with acetone (30 min at −20 °C), blocked with PBS containing 2% BSA and 0.3% TritonX (Sigma-Aldrich) and stained with anti- Ki-67 antibody (clone Mib-1; BD Biosciences, Erembodegem, Belgium) overnight at room temperature. As secondary antibody, biotinylated rabbit-anti-mouse IgG (DAKO) was used, followed by incubation with Streptavidin-FITC (AbD Serotec). DAPI was used to counterstain cell nuclei. Cells were visualized using a fluorescence microscope and appropriate filters (Carl Zeiss).

### Statistical analysis

Graft survival was analyzed using the Kaplan–Meier survival method. The groups’ cell counts were compared using the *t*-test. A p<0.05 was regarded as statistically significant.

## Results

### Endothelial cell loss on rejected corneal allografts (group R)

**Figure 1:**
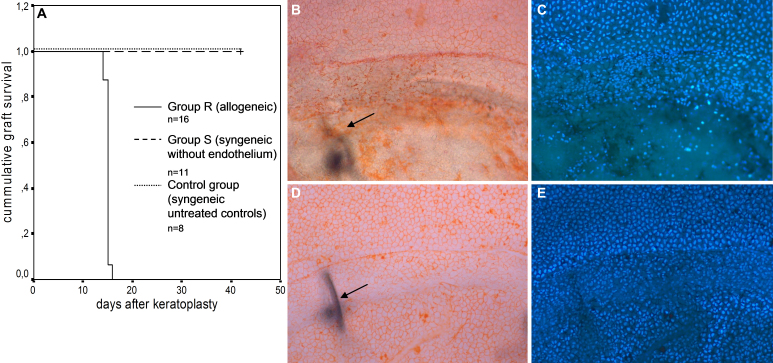
Endothelial cell loss on rejected corneal allografts. **A**: Kaplan–Meier survival analysis: 100% rejection rate of allografts (group R), no rejections for syngeneic controls with endothelium (control group) or following mechanical endothelial removal before syngeneic transplantation (group S). **B**, **C**: Group R: Endothelium at the time of rejection (day 15 after surgery). **B**: Staining with Alicarin red S. **C**: Same cut, DAPI staining of cell nuclei. Superior; Intact host endothelium; Inferior: Graft with endothelium destroyed following immune reaction. Arrow: Suture in the underlying stroma, indicating the graft border. **D**, **E**: Control group: Endothelium of untreated syngeneic controls at the corresponding time point. Superior: Intact host endothelium; Inferior: Intact graft endothelium. Arrow: Suture in the underlying stroma, indicating the graft border.

Allogeneic corneal transplantation was performed between Fisher and Lewis rats (group R). All allografts were rejected after a median time of 15 days following surgery (Figure 1A; n=16). Rejected transplants showed EC loss on the graft site, while the endothelium of the host cornea was fully intact (Figure 1B,C). In contrast, the EC layer was intact at the corresponding time point following syngeneic controls, where no rejection occurred (Figure 1D,E).

### Surgical endothelial abrasion and associated endothelial cell loss (group S)

To further analyze the endothelial response to EC loss following keratoplasty, we introduced a syngeneic model with removal of the graft endothelium before transplantation (group S). These animals showed initially moderate graft opacification, which subsequently decreased. None of the grafts reached an opacity of 4, thus according to our grading system no rejections occurred (Figure 1A; n=11).

**Figure 2:**
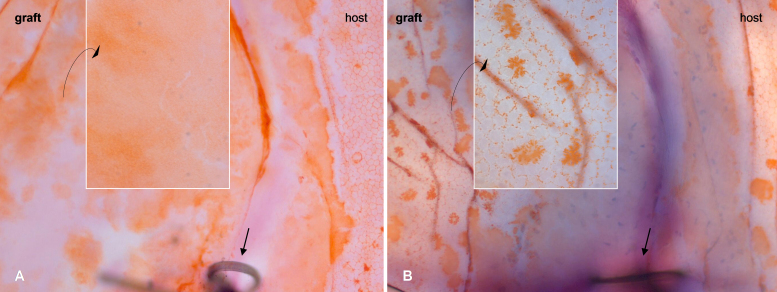
Corneal graft (border zone) on the day of surgery. **A**: Group S: Transplantation of endothelium-free transplant after endothelial debridement. The host endothelium is intact, while the graft is devoid of endothelium (see magnification). **B**: Control group: Transplantation of an untreated graft with endothelium visible on the host cornea and the graft (magnification) directly following surgery. Straight arrow: Suture indicating graft border. Alicarin red S flatmount staining; Magnification: Overview 100×, details 400×.

Alicarin red staining directly following surgical endothelial removal (day 0) confirmed the total absence of graft endothelial cells but intact host endothelium (Figure 2A). Controls that did not undergo endothelial abrasion revealed endothelial cells on the host cornea as well as on the graft immediately following transplantation (Figure 2B). Thus, surgical endothelial removal before syngeneic surgery is an adequate model to examine possible re-endothelialization following keratoplasty without immunological influences.

### Restoration of corneal clarity

The animals of group S were followed clinically for 6 weeks, and those of group R for up to one year after surgery. In both groups, all corneal transplants regained clarity despite prior complete endothelial cell loss.

**Figure 3:**
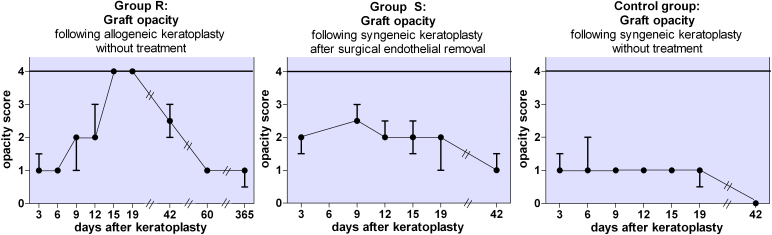
Corneal opacity following keratoplasty. Group R (left): Allogeneic transplantation. Rejection defined as complete graft opacity (opacity grade 4) occurred on average on day 15 after keratoplasty (n=16). All grafts regained clarity in the following weeks to months (follow-up 60 days: n=8, follow-up 1 year: n=8). Group S (middle): syngeneic transplantation of a transplant with surgically removed endothelium. After initially higher opacity compared to the allogeneic group, opacity never reached the maximum grade of 4, thus no rejection occurred (n=11). Control group (right): syngeneic transplantation without prior endothelial alteration. Initial opacity levels are lower compared to the EC-free syngeneic grafts from group S (n=8). (Shown: median±upper/lower quartile).

In group R, all transplants experienced rejection and complete graft opacity approximately two weeks after surgery. About another six weeks later (day 60), the transplants which had previously shown complete graft opacity showed a marked reduction of opacity levels to grade 1. Similarly, in group S, six weeks after syngeneic endothelial cell-free keratoplasty (day 42), the grafts demonstrated a clinical follow-up similar to group R’s with marked reduction of opacity as well (Figure 3). We extended the follow-up of eight of the allogeneic animals to one year. In this time, graft clarity remained stable or further decreased in all grafts.

### Endothelial regeneration

Since the restoration of corneal clarity is only supposed to be possible with a functionally intact EC layer, we performed Alicarin red S stainings counterstained with DAPI on corneal flatmouts to demonstrate corneal ECs on the graft.

**Figure 4:**
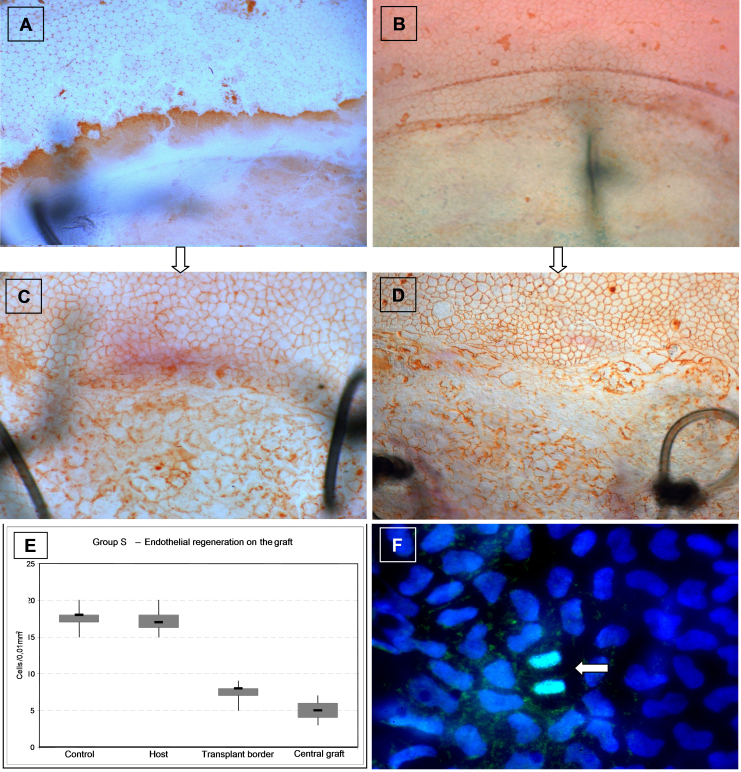
Endothelial regeneration. **A**, **B**: No endothelial cells visible on the grafts, while the host endothelium is intact. **A**: First day after corneal transplantation of an endothelial cell-free syngeneic graft of group S. **B**: Time of rejection in group R. **C**, **D**: Six weeks later newly-formed endothelial cells are visible on the grafts. These cells show an irregular shape and are larger than those in the peripheral recipient endothelium (**C**: Group S; **D**: Group R). **A**-**D**: Superior: Host cornea; Inferior: Graft. Black lines: Sutures indicating the graft border. Magnification 100×. **E**: Following re-endothelialization in group S, mean endothelial cell density on the graft is markedly lower than on the host cornea (Group S, n=11). **F**: Ki-67 (MIB-1) immunostains (green) of a corneal flatmount counterstained with DAPI (blue) shows endothelial cell division (arrow) of host endothelium adjacent to the graft (6 days following surgery). Magnification 630×.

Group S: The denuded graft (Figure 4A) had been repopulated by recipient ECs (Figure 4C) during the weeks following endothelial cell-free keratoplasty. These cells presented pronounced polymorphia and cell enlargement (Figure 4C,E). The area of abnormal endothelial cells extended from the graft onto the adjacent recipient cornea. As shown in Figure 4E, mean EC density was reduced on the graft, whereas no significant changes were apparent on the peripheral host cornea.

Group R: Re-endothelialization also occurred in group R, similarly to group S. While the graft endothelium was destroyed at the time of rejection (Figure 4B), regeneration with a layer of polymorphic ECs occurred on the graft in the following weeks (Figure 4D).

To discover whether the host endothelial cells only expand and migrate onto the graft or additionally also divide and proliferate, we performed Ki-67 stainings on corneal flatmounts. In the area adjacent to the graft and on the graft, but not in the periphery of the host cornea, Ki-67 stainings within the endothelial cell layer revealed scattered EC divisions (Figure 4F). The partner eyes of these animals, which did not undergo surgery, showed an endothelial cell layer which was completely negative for Ki-67. The epithelium of the corneal flatmounts served as internal controls, were cell divisions primarily in the periphery could be detected in all preparations.

### Opacity and immunological infiltration

**Figure 5:**
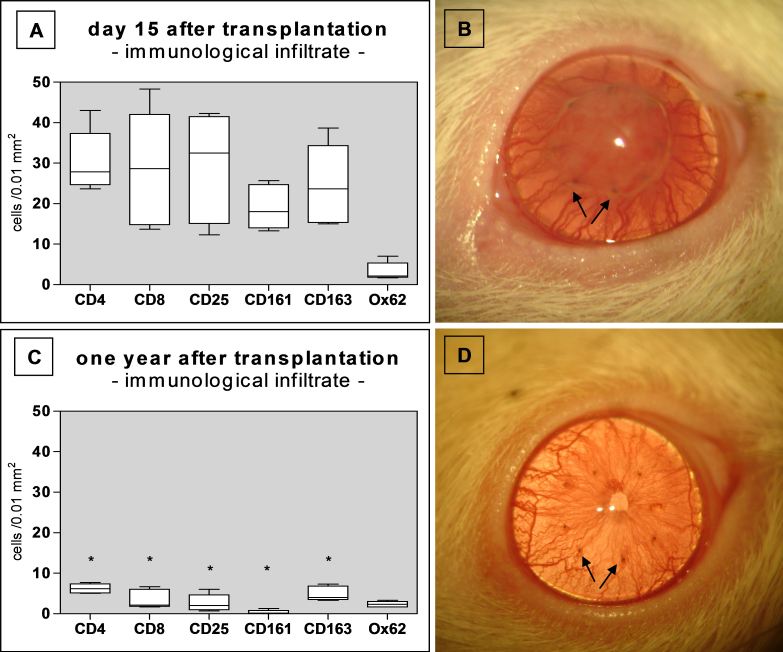
Immune infiltration and corresponding clinical picture (group R). **A**, **B**: Group R: Rate of immune cells infiltrating the graft at the time of rejection (**A**; n=6). At this time point, the graft is completely opaque (**B**). **C**, **D**: Group R: Significant reduction in infiltrating immune cells one year after surgery (**C**; n=6; *p<0.001). Graft clarity is restored (**D**). **A** and **C**: Boxplots. Arrows in **B** and **D** mark sutures indicating the graft border.

Since syngeneic EC-free grafts only achieved opacity scores of 2–3 and did not attain maximum opacity (grade 4) like the allogeneic rejected grafts, immunological factors – aside from a damaged EC layer – must be responsible for opacity grades of 4. Therefore, the immunological infiltrate was analyzed in corneal allografts at the rejection time point and one year following PKP (Figure 5).

At the time of rejection, all allografts showed strong infiltration of CD45^+^ leukocytes. Further immunohistochemical analyses revealed a pronounced infiltrate with T-cells (CD4^+^, CD8^+^), CD25^+^ cells, CD161^+^ NK cells, and CD163^+^ macrophages, whereas few dendritic cells (OX62^+^) were present (Figure 5A). A clinical example of a rejected and infiltrated graft is illustrated in Figure 5B. One year following complete rejection, the cellular infiltrate was significantly reduced, except for dendritic cells (Figure 5C). This correlated with a clinically clear allograft as shown in Figure 5D.

## Discussion

The rat keratoplasty model is a widely used model for corneal transplantation [21]. Allogeneic corneal transplantation using adult rats of Fisher strain as donors and adult Lewis rats as recipients leads to a 100% rejection rate and is thus is a reliable model for studying immunological responses [22,23]. A rejected graft shows complete corneal opacification, and in most studies, the animals are sacrificed at this time point. In contrast, we have extended the follow-up time up to one year following corneal transplantation, and noted that all transplants that had undergone rejection regained clarity.

Allogeneic corneal transplantation with subsequent rejection is thought to be associated with permanent EC loss in both humans [24,25] and rodent models of penetrating keratoplasty [26,27]. However, we observed that the rejected grafts cleared up again. This would require a functioning endothelial cell layer on the graft. In fact, we observed a loss of endothelial cells due to the immunological response, with an almost complete absence of endothelial cells following rejection, and subsequent re-endothelialization in the following weeks to months.

However, very little is known about recovery from opacification or re-endothelialization following keratoplasty in the rat. Williams and Coster, who introduced a penetrating rat keratoplasty model in 1985 with Fisher rats as recipients and DA rats as donors, described that, out of 21 allografts to prevascularized corneal beds (all of which became “thick and cloudy” within 12 days postgraft), 5 regained clarity 3 to 4 weeks following keratoplasty. However, since they provided neither grades of opacity nor exact time responses, it is difficult to conclude whether these 5 animals had truly rejected their graft. They did not assess the endothelium in their follow-up investigation [21].

Only Gong et al. [19] have so far provided a detailed description of corneal clarity restoration in all grafts following keratoplasty between Dark Agouti rats as donors and Lewis rats as recipients. In their model, an opacity grade of 3 was equalized with rejection, leading to a rejection rate of 100%, followed by the clearing of all grafts over the following three weeks. Endothelial cells were not stained specifically but recognized via nuclear morphology of corneal buttons. These showed the absence of cells at the time of rejection, and a continuous increase in cells in the following weeks. In our experiments, ECs were stained using Alicarin red S to display the endothelial cell borders. In combination with DAPI staining of the cell nucleus, this provides a more detailed characterization of EC morphology and distribution compared to previous studies.

Two models of re-endothelialization have been suggested in the literature: the survival of donor endothelial cells with subsequent functional recovery, or the regeneration of an endothelial cell layer by the host endothelium. Plskova et al. [18] postulated that donor endothelial cells survive the immune response and regain their function. In their mouse model, allogeneic keratoplasty lead to a variable degree of graft opacity, with rejection defined as graft opacity greater than 2. While a clinically assumed rejection was mostly associated with endothelial cell loss, the grade of graft opacity did not accurately reflect the degree of endothelial cell coverage. Those authors described endothelial cell migration and cell enlargement, but felt that there was no evidence of replacement by host endothelium [26]. A study by Hori et al. [27] also demonstrated endothelial cell loss due to rejection in a GFP mouse model, but unlike other authors, they did not observe subsequent re-endothelialization. Accepted grafts (all syngeneic and several allogeneic grafts) kept their endothelium. They thus concluded that recipient endothelium could not overgrow the donor corneal button. Their findings contrast strongly to our observations in this study. In our experiments, we noted re-endothelialization following rejection, as well as after mechanical abrasion. Mechanical abrasion guaranteed the use of completely EC-free transplants in group S. These experiments enabled us to demonstrate that recipient endothelium is capable of extending over the perforating scar of the transplant edge, and capable of covering the grafted area. Moreover, we demonstrated that these endothelial cells – although morphologically altered - retain their functional capacity to restore corneal clarity. Analogously, we suggest a similar pattern of host-derived endothelial regeneration in our allogeneic transplants of group R. Although we did not prove the origin of the endothelial cells in this set of experiments, this hypothesis could be further tested by grafting experiments to GFP^+^ recipient rats or by sex-mismatch operations, followed by analysis of the regenerated allograft endothelium for GFP-expression or for sex chromatin expression, respectively.

At the time of rejection, a strong immunological infiltration with a variety of leukocytes (Figure 5A) coincides with complete EC loss (Figure 1B,C). The combination of both leads to complete graft opacity, defined as graft rejection. In contrast, the EC loss without immune response in group S with endothelial abrasion resulted only in a relative opacity of ≤grade 3. Thus, since a clinically-apparent grade 3 can be achieved without an allogeneic immune response, we recommend using only complete graft opacity (grade 4) as the clinical rejection criterion in this rat model.

Gong et al. [19] described re-endothelialization of corneal allografts in the rat. We not only can confirm their observation, we are the first to also analyze the role of the immune reaction in this context. The immunological infiltration we observed throughout the graft at the time of rejection (Figure 5A) later subsides (Figure 5C). Analogous to the coincidence of maximum immune cell infiltration, endothelial cell loss, and complete opacity at the time of rejection, the subsequent reduction in leukocyte infiltration coincides with restored corneal clarity. Since the endothelium is thought to be the major target for an immunological allo-response, we hypothesize that, following complete rejection, the immune reaction subsides due to destruction of the allogeneic cellular material. Corneal clarity may thereafter be restored by syngeneic host corneal endothelium that does not provoke further immune stimulation. However, as mentioned above, to prove this theory it would be necessary to directly determine EC origin, e.g., by using GFP transgenic animals or detecting Y-chromosomes in sex mismatch PKP settings.

We also investigated EC recovery mechanisms. Loss of contact inhibition due to the large defect in the EC layer might be the stimulus for the cells to migrate and enlarge. EC proliferation has also been hypothesized in this context. However, that was based on the morphological arrangement of cell nuclei, and nothing has been reported on specific proliferation markers in this context so far [19]. In our set of experiments, we demonstrated Ki-67^+^ -cells of the endothelial cell layer on corneal flatmounts. This antigen is expressed during all active phases of the cell cycle and thus acts as a widely-used proliferation marker [28]. We detected dividing cells close to the graft border (Figure 4F), a finding that supports observations made by Tuft et al., who found endothelial cell divisions at the margin of corneal endothelial wounds in rats [14]. Without an EC wound’s stimulus, EC division does not occur in the adult rat [29]. Dividing ECs in vivo have been found in animals other than the rat that have been used in corneal experiments like rabbit [15,30], rhesus monkeys [15], and cats [16,17]. Even though in vitro assays and experiments with cultured corneas suggested a higher incidence of endothelial precursor cells in the corneal periphery [13,31], we only observed Ki-67^+^ cells in the endothelial cell layer close to the graft border and not in the periphery of the host corneas.

In summary, our experiments demonstrate that in vivo re-endothelialization exists following PKP in the rat. The host endothelium is capable of repopulating an EC-free graft with functional endothelium. This seems to occur by a combination of cell enlargement and cell shift from the host toward the graft, and by local cell division. Following a rejection episode which destroys the corneal endothelium, the immune reaction subsides, and the number of infiltrating cells diminishes dramatically over the following weeks and months. The formerly opaque graft regains its clarity, and endothelial cells re-cover the graft. Even though the new endothelial cell layer is morphologically altered, it seems sufficient to restore graft clarity.
